# Evidence that resistin acts on the mechanical responses of the mouse gastric fundus

**DOI:** 10.3389/fphys.2022.930197

**Published:** 2022-07-15

**Authors:** Eglantina Idrizaj, Rachele Garella, Silvia Nistri, Roberta Squecco, Maria Caterina Baccari

**Affiliations:** ^1^ Department of Experimental and Clinical Medicine, Section of Physiological Sciences, University of Florence, Florence, Italy; ^2^ Department of Experimental and Clinical Medicine, Research Unit of Histology and Embryology, University of Florence, Florence, Italy

**Keywords:** resistin, neuromodulation, nitric oxide, gastric motility, satiety signals

## Abstract

Resistin, among its several actions, has been reported to exert central anorexigenic effects in rodents. Some adipokines which centrally modulate food intake have also been reported to affect the activity of gastric smooth muscle, whose motor responses represent a source of peripheral signals implicated in the control of the hunger-satiety cycle through the gut-brain axis. On this basis, in the present experiments, we investigated whether resistin too could affect the mechanical responses in the mouse longitudinal gastric fundal strips. Electrical field stimulation (EFS) elicited tetrodotoxin- and atropine-sensitive contractile responses. Resistin reduced the amplitude of the EFS-induced contractile responses. This effect was no longer detected in the presence of L-NNA, a nitric oxide (NO) synthesis inhibitor. Resistin did not influence the direct muscular response to methacholine. In the presence of carbachol and guanethidine, EFS elicited inhibitory responses whose amplitude was increased by resistin. L-NNA abolished the inhibitory responses evoked by EFS, indicating their nitrergic nature. In the presence of L-NNA, resistin did not have any effect on the EFS-evoked inhibitory responses. Western blot and immunofluorescence analysis revealed a significant increase in neuronal nitric oxide synthase (nNOS) expression in neurons of the myenteric plexus following resistin exposure. In conclusion, the present results offer the first evidence that resistin acts on the gastric fundus, likely through a modulatory action on the nitrergic neurotransmission.

## 1 Introduction

White adipose tissue is nowadays considered an endocrine organ, being able to produce and release a variety of hormones called adipokines (Rodríguez et al., 2015). These latter include resistin, a polypeptide secreted by mammals, belonging to a family of proteins called resistin-like molecules that comprises several members with distinct patterns of expression and biological actions (Steppan et al., 2001). Resistin was originally claimed to be adipose-specific, since it was found to be primarily expressed and secreted by mature white adipocytes of rodents (Steppan et al., 2001). Subsequently, its expression in rodents has been revealed in multiple other structures including the hypothalamus (Morash et al., 2002) and the gastrointestinal tract (Nogueiras et al., 2003) as well as in various human cells (Recinella et al., 2020; Badoer, 2021).

Several potential resistin receptors have been suggested but its specific one has not yet been identified and scarce information concerning the downstream signaling mechanisms of action is available (Badoer, 2021). Nevertheless, the hormone has been reported to be involved in a broad range of physiological and pathological conditions, both in rodents and in humans, acting centrally and peripherally (Aitken-Buck et al., 2020; Recinella et al., 2020; Deb et al., 2021; Rachwalik et al., 2021). In particular, resistin acting at the hypothalamic level influences energy homeostasis and modulates feeding, displaying anorexigenic effects in rodents (Brunetti et al., 2004; Tovar et al., 2005; Vázquez et al., 2008; Cifani et al., 2009). Central administration of resistin has indeed been reported to promote short-term satiety in rats (Tovar et al., 2005), and the effects of the hormone were found to be associated with changes in mRNA expression of neuropeptides with orexigenic and anorexigenic properties in the hypothalamic arcuate nucleus (Vázquez et al., 2008). In this view, a reduced hyperphagic effect of neuropeptide Y by resistin has also been observed in rats, confirming the involvement of the hormone in the central regulation of feeding behavior (Cifani et al., 2009). This regulation also involves peripheral hormonal and nervous signals from the gastrointestinal tract (Camilleri, 2015; Moura-Assis et al., 2021; Tack et al., 2021). In particular, gastric motor phenomena are known to generate nervous signals which reach the hypothalamic nuclei implicated in the control of the hunger-satiety cycle through the gut-brain axis (Janssen et al., 2011).

Notably, some substances which centrally influence feeding have also been reported to affect motor responses of the stomach (Guan et al., 2012; Idrizaj et al., 2019b; Camilleri, 2019; Goyal et al., 2019; Idrizaj et al., 2020b). The latter are indeed controlled by a variety of hormones, some of which act by modulating the release of neurotransmitters (Camilleri, 2019; Verbeure et al., 2021; Baccari et al., 2022). Among these, nitric oxide (NO) appears to be one of the main molecules whose release/production is influenced by this hormonal control (Idrizaj et al., 2021; Verbeure et al., 2021).

NO is considered as the major inhibitory neurotransmitter released from non-adrenergic, non-cholinergic (NANC) fibers supplying the smooth muscle and responsible for proximal stomach relaxation in both humans and rodents (Rand, 1992; Min et al., 2016; Idrizaj et al., 2021). In this view, we previously observed that a NO-dependent mechanism was implicated in the inhibitory actions of adiponectin on gastric smooth muscle activity (Idrizaj et al., 2018a; Idrizaj et al., 2019a; Idrizaj et al., 2020a). Interestingly, NO production has been shown to be also influenced by resistin in human vascular endothelial cells (Chen et al., 2010).

All the above observations, together with the recent finding that adiponectin and resistin are co-secreted by white mouse adipocytes (Musovic et al., 2021), have stimulated our interest in examining whether resistin too could have an action at the gastric level since, to our knowledge, there are no studies in the literature on this topic. Therefore, we here investigated the effects of resistin on the mechanical responses in longitudinal preparations from the mouse gastric fundus and the possible involvement of NO through a combined functional and immunohistochemical approach.

## 2 Materials and methods

Experiments were conducted on female mice (C57BL/6; Charles River, Lecco, Italy) 8- to 12-week-old. The animals, fed with standard laboratory chow and water, were housed at a controlled temperature (21 ± 1°C) and under a 12-h light/12-h dark photoperiod. The experimental protocol was designed in accordance with the guidelines of the European Communities Council Directive 2010/63/UE and the recommendations for the care and use of laboratory animals approved by the Animal Care Committee (University of Florence, Italy), subject to the authorization of the Italian Ministry of Health (code 0DD9B.N.ZB6/2020 to MCB). The animals were sacrificed by cervical dislocation.

### 2.1 Mechanical recording

As previously reported (Vannucchi et al., 2011; Garella et al., 2017a; Idrizaj et al., 2018a), the stomach was quickly removed from the abdomen and full-thickness strips (2 × 10 mm) were cut in the direction of the longitudinal muscle layer from the fundal region. One end of each strip was tied to a platinum rod, while the other was connected to a force displacement transducer (Grass model FT03, Quincy, MA, USA) by a silk thread for continuous recording of isometric tension. The transducer was coupled to polygraph systems (Grass model 7K, Quincy, MA, USA). Preparations were mounted in the longitudinal direction in 5 ml double-jacketed organ baths containing Krebs-Henseleit solution, gassed with 95% O_2_-5% CO_2_ mixture, of the following composition (mM): NaCl 118, KCl 4.7, MgSO_4_ 1.2, KH_2_PO_4_ 1.2, NaHCO_3_ 25, CaCl_2_ 2.5 and glucose 10 (pH 7.4). The temperature was maintained within a range of 37 ± 0.5°C.

Electrical field stimulation (EFS) was applied via two platinum wire rings (2 mm diameter, 5 mm apart) through which the preparation was threaded. Electrical pulses (rectangular waves, 80 V, 4 and 8 Hz, 0.5 ms, for 15 s) were provided by a Grass model S8 stimulator. Strips were allowed to equilibrate for 1 h under an initial load of 0.8 g.

### 2.2 Western blotting and immunofluorescence analysis

Gastric specimens were employed. One-half of each gastric fundus was exposed to resistin (60 ng/ml) for 30 min whereas the second half was maintained in Krebs-Henseleit solution for the same time without adding the hormone (controls), and then preparations were immediately processed for western blot and immunohistochemistry.

#### 2.2.1 Western blotting

Fragments of gastric fundus from the control and resistin exposed specimens were homogenized in cold lysis buffer (20 mM Tris/HCl (pH 7.4), 10 mM NaCl, 1.5 mM MgCl_2_, 5 mM EGTA, 2 mM Na_2_EDTA, added with 10x Sigmafast Protease Inhibitor Cocktail tablets). Total protein content was measured spectrophotometrically using micro-BCA™ Protein Assay Kit (Pierce, IL, USA). Fifty μg of total proteins were electrophoresed by SDS–PAGE and blotted onto PVDF membranes (Millipore, Bedford, MA, USA). The membranes were incubated overnight (O.N.) at 4 °C with rabbit polyclonal anti-nNOS (1: 2000; Millipore) and mouse monoclonal anti-GAPDH (1:2000; Invitrogen, Waltham, Massachusetts, USA), assuming GAPDH as control invariant protein. Specific bands were detected using rabbit peroxidase-labeled secondary antibodies (1:15.000 Vector, Burlingame, CA, USA) and enhanced chemiluminescent substrate. Densitometric analysis of the bands was performed using Scion Image Beta 4.0.2 image analysis software (Scion Corp., Frederick, MD, USA).

#### 2.2.2 Immunofluorescence analysis

Gastric tissue samples were fixed in 4% paraformaldehyde, embedded in paraffin, and cut into 5-μm thick sections. For antigen retrieval the sections were transferred in EDTA 1 mM, pH 9.0 + tris buffer 10 mM, for 20 min at a temperature of 90–92°C. To quench the autofluorescence of elastic fibers, the sections were incubated in 2 mg/ml glycine for 8 min at room temperature (RT). To minimize the unspecific binding, the sections were pre-incubated with 1.5% bovine serum albumin (Sigma Aldrich) for 20 min at RT and then incubated O.N. at 4°C with rabbit monoclonal anti-nNOS antibody (1:2000, Millipore) followed by goat anti-rabbit Alexa Fluor 488-conjugated IgG (1:350 Invitrogen) for 2 h at RT. After the first incubation as described above, the sections were re-incubated O.N. at 4°C with anti-mouse monoclonal anti-UCH-L1 (neuronal marker) antibody (1:200; Santa Cruz Biotechnology, Texas, USA). Then, the sections were incubated with the appropriate Alexa Fluor 568-conjugated IgG (1:350; Invitrogen) for 2 h at RT. Negative controls were performed by omitting the primary antibodies. The sections were mounted with FluoroshiedTM mounting medium containing the nuclear marker DAPI (Sigma Aldrich). Images were obtained using an epi-fluorescence Olympus BX40 microscope coupled to analySIS∧B Imaging Software (Olympus, Milan, Italy) with a ×40 objectives.

### 2.3 Drugs

Atropine, guanethidine sulphate, carbachol (CCh), mouse recombinant resistin, methacholine, tetrodotoxin (TTX), and L-N^G^-nitro arginine (L-NNA) were obtained from Sigma Chemical (St. Louis, MO, United States). Drug concentrations are in the range of those previously reported to be effective in isolated smooth muscle tissues (Scott et al., 2016; Idrizaj et al., 2018a; Wang et al., 2021).

### 2.4 Data analysis

Amplitude of contractile responses is given as percentage of the muscular contraction elicited by 2 × 10^−6^ M methacholine, taken as 100%, and measured 30 s after a stable plateau phase was reached. The basal tension value was assumed as 0 g, since baseline was regained following the equilibration period. Relaxant responses are expressed as a percentage decrease relative to the muscular tension induced by 1 × 10^−6^ M CCh just before obtaining relaxations. Amplitude values of EFS-induced relaxations refer to the maximal peak obtained during the stimulation period.

### 2.5 Statistical analysis

The statistical significance was assessed by paired or unpaired Student’s *t*-test or one-way ANOVA followed by Bonferroni’s *post hoc* test for multiple comparisons. A *P* value <0.05 was considered significant. Results are means ± SEM. In the results *n* indicates the number of preparations.

## 3 Results

### 3.1 Resistin decreases the amplitude of the neurally-evoked excitatory responses

**FIGURE 1:**
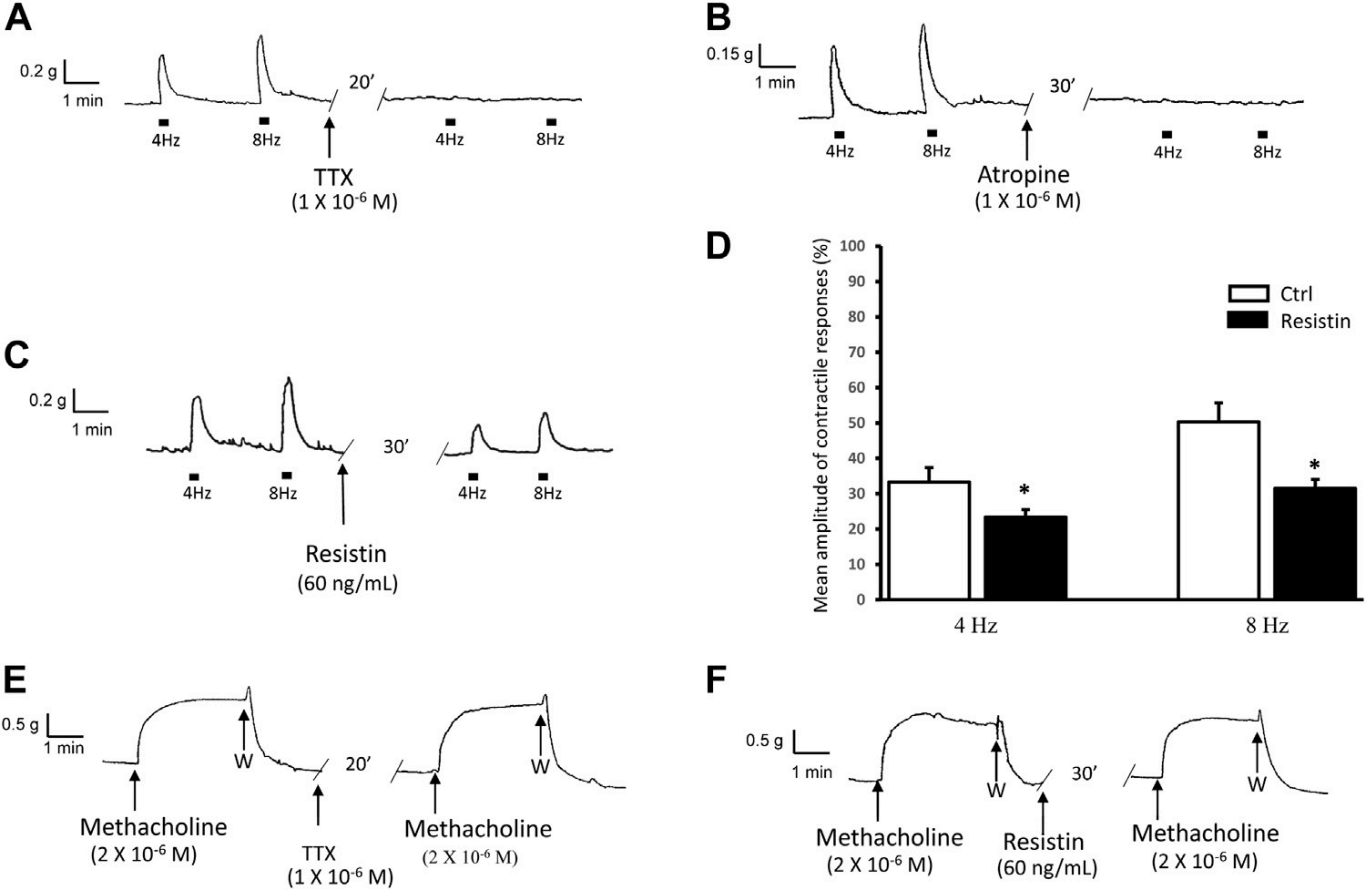
Influence of resistin on the neurally-evoked contractions and its lack of effects on the response to methacholine. **(A,B)**: representative traces showing the EFS-evoked contractile responses at 4 and 8 Hz stimulation frequency (left hand traces) and their abolition in the presence of TTX [**(A)**, right hand trace] or atropine [**(B)**, right hand trace]. **(C,D)**: representative trace **(C)** showing that the amplitude of the neurally-induced excitatory responses at both stimulation frequencies employed (left hand trace) is decreased in the presence of 60 ng/ml resistin (right hand trace). Bar chart **(D)** of the effects of resistin on the mean amplitude of the EFS-evoked contractions at 4 and 8 Hz stimulation frequency. Amplitude of contractile responses is expressed as percentage of the muscular contraction induced by 2 × 10^−6^ M methacholine, taken as 100%. All values are means ± SEM of 8 strips (from 5 animals). *p < 0.05 *vs* Ctrl (Student’s *t*-test). **(E,F)**: Representative traces showing the response to methacholine (left hand traces), whose amplitude is not affected by TTX [**(E)**, right hand trace] or resistin [**(F)**, right hand trace].

As previously observed (Garella et al., 2017a; Idrizaj et al., 2018a), at basal tension, EFS (4–8 Hz) evoked (*n* = 28) excitatory responses which were abolished by 1 × 10^−6^ M TTX (*n* = 2, from 2 mice) or 1 × 10^−6^ M atropine (*n* = 2, from 2 mice) (Figures 1A,B), indicating their nervous and cholinergic nature, respectively.

Addition of resistin (60 ng/ml) to the bath medium (*n* = 14, from 7 mice) decreased the amplitude of the contractile responses induced by EFS (Figures 1C,D). The effects of resistin were already detectable 15–20 min following its inclusion into the bath medium and lasted for 1 h (no longer time recorded).

Addition of methacholine (2 × 10^−6^ M) to the bath medium (*n* = 8, from 5 mice) caused a sustained contracture which was TTX-insensitive (*n* = 2, from 2 mice) and reached a plateau phase (mean amplitude 1.0 ± 0.2 g) that lasted until washout (Figure 1E). Resistin (60 ng/ml) did not influence (*n* = 6 from 3 mice) the amplitude of the direct muscular response to methacholine (mean amplitude 1.1 ± 0.1 g, *p* > 0.05), as shown in Figure 1F.

### 3.2 L-NNA counteracts the effects of resistin on the neurally-evoked excitatory responses

**FIGURE 2:**
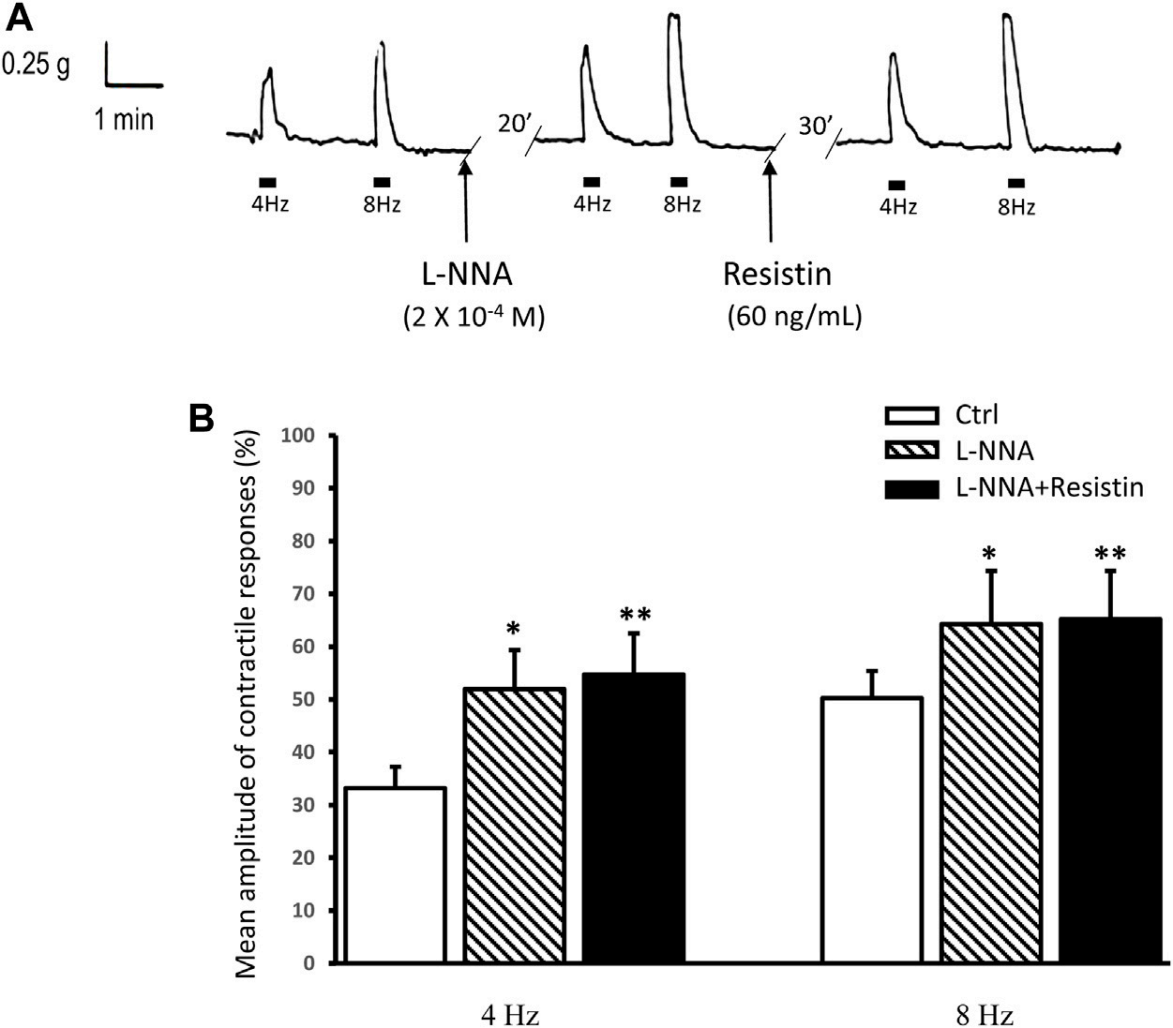
Lack of effects of resistin on the neurally-evoked contractile responses in the presence of L-NNA. **(A)**: representative trace showing the EFS-evoked contractile responses at 4 and 8 Hz stimulation frequency (left hand panel). Addition of 2 × 10^−4^ M L-NNA to the bath medium enhances the amplitude of the neurally-evoked contractile responses (middle panel). In the presence of L-NNA, resistin (60 ng/ml) no longer depresses the amplitude of the EFS-evoked contractile responses (right hand panel). **(B)**: bar chart showing the lack of effects of resistin on the mean amplitude of the neurally-evoked contractile responses in the presence of L-NNA. Amplitude of contractile responses is expressed as percentage of the muscular contraction induced by 2 × 10^−6^ M methacholine, taken as 100%. All values are means ± SEM of 8 strips (from 4 animals). *p < 0.05 L-NNA *vs* its own Ctrl; **p < 0.05 L-NNA + resistin *vs* its own Ctrl and *p* > 0.05 L-NNA + resistin *vs* L-NNA (ANOVA with Bonferroni’s post hoc test).

Addition of the NO synthesis inhibitor L-NNA (2 × 10^−4^ M) to the bath medium (*n* = 8, from 4 mice) did not cause appreciable changes of the strip basal tension but enhanced the amplitude of the EFS-induced contractions (Figure 2). In the presence of L-NNA, resistin (60 ng/ml) no longer decreased (in all the 8 strips) the amplitude of the contractile responses evoked by EFS (*p* > 0.05; Figures 2A,B).

### 3.3 Resistin increases the amplitude of the neurally-evoked relaxant responses

The effects of resistin were investigated in the presence of guanethidine (1 × 10^−6^ M) and CCh (1 × 10^−6^ M).

**FIGURE 3:**
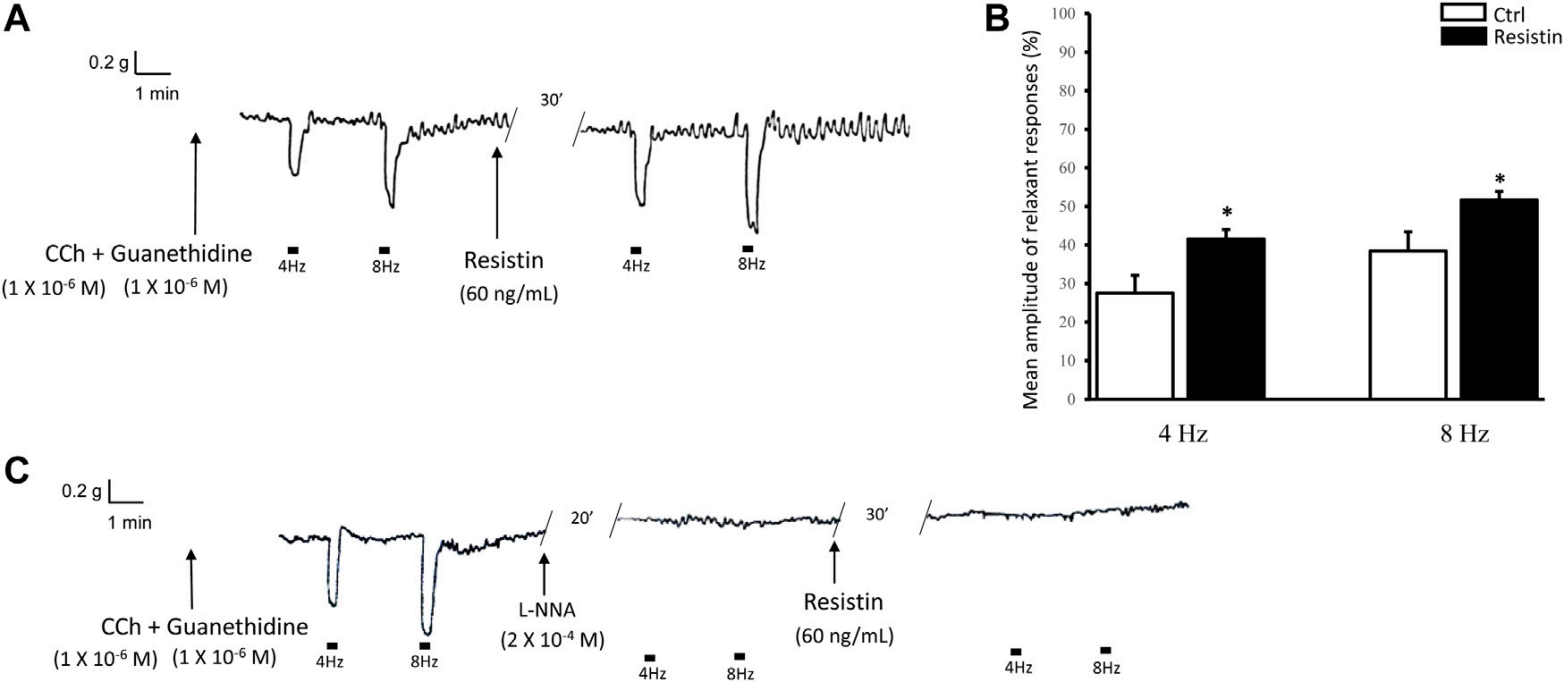
Influence of resistin on the neurally-evoked relaxant responses and its lack of effects in the presence of L-NNA. **(A,B)**: representative trace showing that the EFS-evoked relaxant responses at 4 and 8 Hz stimulation frequency [**(A)**, left hand trace] are increased in amplitude in the presence of 60 ng/ml resistin (right hand trace). Bar chart **(B)** of the effects of resistin on the mean amplitude of the EFS-induced relaxant responses. Amplitude values refer to the maximal peak obtained during the stimulation period and represent percentage decreases relative to the muscular tension induced by CCh (1 × 10^−6^ M), taken as 100%. All values are means ± SEM of 8 strips (from 4 mice). *p < 0.05 *vs* Ctrl (Student’s t-test). **(C)**: Representative tracing showing that the EFS-evoked inhibitory responses at 4 and 8 Hz stimulation frequency (left hand trace) are abolished by 2 × 10^−4^ M L-NNA (middle trace) and the subsequent addition of resistin (60 ng/ml) to the bath medium has no longer effects (right hand trace) on the neurally-evoked relaxant responses. Note the absence of responses to EFS at 4 and 8 Hz stimulation frequency in the presence of either L-NNA or L-NNA + resistin.

CCh, added to the bath medium (*n* = 16), caused a rapidly arising contraction (mean amplitude 1.2 ± 0.2 g), and after washout strip tension returned to baseline. In CCh precontracted strips, EFS (4 and 8 Hz) elicited a fast relaxant response which persisted during the whole period of stimulation (Figure 3A). Addition of resistin (60 ng/ml) to the bath medium (*n* = 12, from 6 mice) elicited, in 8 out of the 12 strips, a slight and lasting decay of the basal tension (mean amplitude 7.0 ± 0.2%; *p* < 0.05). In the presence of resistin the amplitude of the EFS-evoked relaxation was increased (*p* < 0.05; Figures 3A,B). The effects of resistin were already detectable 15–20 min after its inclusion into the bath medium and lasted for 1 h (no longer time recorded).

In the presence of L-NNA (2 × 10^−4^ M), the EFS-evoked relaxant responses were abolished (*n* = 4, from 3 mice) and the subsequent addition of resistin (60 ng/ml) to the bath medium had no longer effects (Figure 3C).

### 3.4 Resistin increases nNOS expression

**FIGURE 4:**
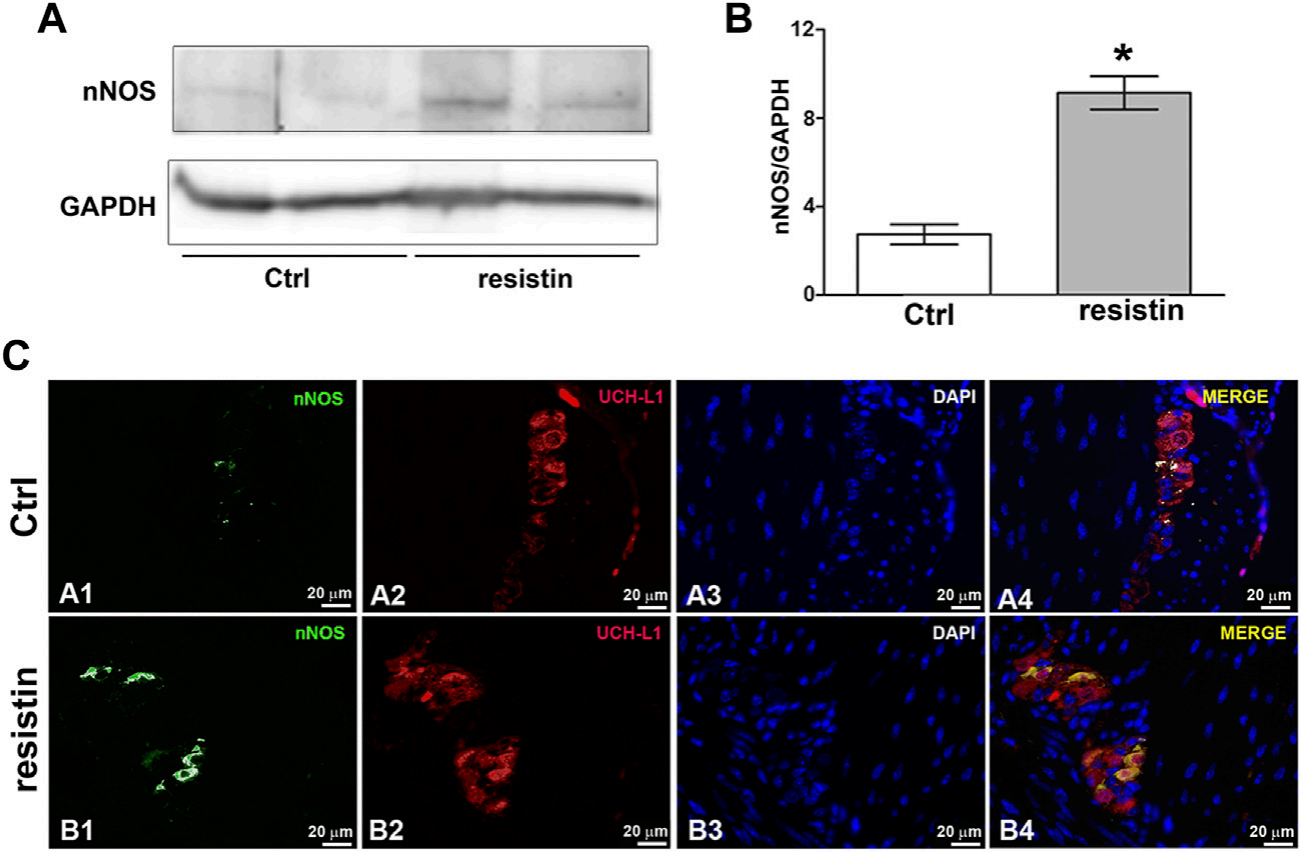
Resistin up-regulates nNOS expression. **(A)** Effect of resistin exposure (30 min) on nNOS expression in mouse gastric fundus assayed by Western blotting: representative bands from a typical experiment. The two representative lanes for control and resistin samples are biological replicates from separate animals. **(B)** Quantitative analysis. Columns are means ± SEM. Significance of differences (Student’s t-test for two independent samples from 3 mice): *p < 0.05 *vs* controls (Ctrl). **(C)** Representative photomicrographs of gastric tissue from control and resistin-exposed (30 min) preparations showing double immunofluorescence labeling. (A1–A4) Co-localization of nNOS and UCH-L1 in control mice: (A1) nNOS signal (green channel); (A2) UCH-L1 signal (red channel); (A3) DAPI; (A4) merged images. (B1–B4) Co-localization of nNOS and UCH-L1 in resistin-exposed preparations: B1 nNOS signal (green channel); (B2) UCH-L1 signal (red channel); (B3) DAPI; (B4) merged images. Scale bar: 20 μm.

Exposure to resistin for 30 min induces a significant increase in nNOS expression in the mouse gastric fundus as demonstrated by western blot (ctrl 2.750 ± 0.45; resistin 9.150 ± 0.75; *p* < 0.05) and immunofluorescence analysis that showed nNOS expression in the neurons of the myenteric plexus (Figure 4).

## 4 Discussion

The main finding of this study provides the new information that resistin is capable of acting on the mouse gastric fundus. In particular, the hormone appears to affect the mechanical responses mainly through a neuromodulatory action on the nitrergic transmission.

Resistin indeed influenced the amplitude of the neurally-evoked both excitatory and inhibitory responses in longitudinal strips from the mouse gastric fundus. In particular, resistin decreased the amplitude of the neurally-induced excitatory responses without affecting the direct smooth muscle contraction to methacholine. This observation, other than excluding a non-specific effect, indicates that the hormone exerts a neuromodulatory action. However, gastric motor responses are known to be the result of a balance between excitatory (mainly cholinergic) and inhibitory (NANC) nervous influences on the smooth muscle. Therefore, being both types of nerve fibers activated during EFS, the decrease in amplitude of the neurally-evoked contractile responses by resistin could be due to either a reduced activation of the excitatory component or to an increased activation of the inhibitory one. In the present experiments, the ability of L-NNA to increase the amplitude of the EFS-evoked contractile responses indicates the removal of a nitrergic inhibitory influence at the nervous level. Notably, the observation that resistin no longer influenced the amplitude of the neurally-evoked excitatory responses in the presence of L-NNA, suggests that the hormone exerts a neuromodulatory action on the nitrergic transmission. This is further confirmed by the observation that, in the presence of L-NNA, resistin was no longer able to increase the amplitude of the EFS-evoked inhibitory responses, whose abolition by L-NNA also supports that NO is the inhibitory neurotransmitter released during EFS in gastric fundal preparations from mice (Mulè and Serio, 2003; Baccari et al., 2009; Garella et al., 2017a; Idrizaj et al., 2018a).

In agreement, we revealed a significant increase in nNOS expression in neurons of the myenteric plexus following resistin exposure. The nNOS isoform, producing NO as a neurotransmitter which indeed plays the most important role in the control of gastrointestinal motility (Groneberg et al., 2016) appears as a shared target for many hormones (Garella et al., 2017a,b; Bódi et al., 2019; Idrizaj et al., 2021; Baccari et al., 2022).

NO released from inhibitory enteric motor neurons acts on SIP syncytium cells (smooth muscle cells, interstitial cells of Cajal, and PDGFRα+ cells) (Blair et al., 2012) and it could be not excluded that a direct effect of resistin, in addition to its modulatory action on the nitrergic neurotransmission, may occur at the muscular level through the involvement of different NO pathways on SIP cells. In this view, the effects of resistin might enhance the action of NO at the muscular level. This can likely occur through the sGC/cGMP pathway, as observed for other hormones (Squecco et al., 2015; Idrizaj et al., 2018b), or even by decreasing NO-induced effects on Ca^2+^ sensitization proteins (Sanders and Ward, 2019) which may be in accordance with the observation that the hormone also causes a slight decay of the basal tension. This topic certainly deserves to be better investigated.

Different mechanisms of action have been indeed reported to be engaged by resistin also on causing vasoconstriction. In this view, an enhancement of calcium entry from SOC in vascular smooth muscle cells has also been reported (Chuang et al., 2012). Moreover, the pro-inflammatory adipokine resistin appears to cause vasoconstriction mainly impairing endothelial functions due to increased endothelin-1 production and decreased endothelial NOS expression and NO levels (Maenhaut and Van de Voorde, 2011). Therefore, NO seems to be involved in resistin actions also on the vascular smooth muscle.

The results of the present study offer the first evidence that resistin is capable of acting on the gastric fundus, at least in part, through a modulatory action on the nitrergic neurotransmission.

## Data Availability

The original contributions presented in the study are included in the article/Supplementary Material, further inquiries can be directed to the corresponding authors.

## Abbreviations

Ctrl, control; EFS, electrical field stimulation; L-NNA, L-N^G^-nitro arginine; NANC, non-adrenergic, non-cholinergic; NO, nitric oxide; NOS, nitric oxide synthase; nNOS, neuronal nitric oxide synthase.
